# 1,3-Bis(thio­phen-2-ylmeth­yl)-3,4,5,6-tetra­hydro­pyrimidinium trichlorido(η^6^-*p*-cymene)ruthenate(II)

**DOI:** 10.1107/S1600536808042773

**Published:** 2008-12-20

**Authors:** Hakan Arslan, Don VanDerveer, İsmail Özdemir, Nevin Gürbüz, Yetkin Gök, Bekir Çetinkaya

**Affiliations:** a Department of Natural Sciences, Fayetteville State University, Fayetteville, NC 28301, USA; b Department of Chemistry, Faculty of Pharmacy, Mersin University, Mersin, TR 33169, Turkey; c Department of Chemistry, Clemson University, Clemson, SC 29634, USA; d Department of Chemistry, Faculty of Science and Arts, İnönü University, Malatya, TR 44280, Turkey; e Department of Chemistry, Faculty of Science, Ege University, Bornova-İzmir, TR 35100, Turkey

## Abstract

The asymmetric unit of the title compound, (C_14_H_17_N_2_S_2_)[Ru(C_10_H_14_)Cl_3_], contains a 1,3-bis­(thio­phen-2-ylmeth­yl)-3,4,5,6-tetra­hydro­pyrimidinium cation and a trichlorido(η^6^-*p*-cymene)ruthenate(II) anion. The Ru atom exhibits a distorted octa­hedral coordination with the benzene ring of the *p*-cymene ligand formally occupying three sites and three chloride atoms occupying the other three sites. The N—C bond lengths of the N—C—N unit of the pyrimidinium cation are shorter than the average single C—N bond length of 1.48 Å, thus showing double-bond character, indicating a partial electron delocalization within the N—C—N fragment. The pyrimidine ring has an envelope conformation. Four inter­molecular C—H⋯Cl hydrogen bonds generate a three-dimensional hydrogen-bonded framework.

## Related literature

For the synthesis, see: Yaşar *et al.* (2008 ▶); Özdemir *et al.* (2005*a* ▶, 2005*b* ▶, 2007 ▶, 2008 ▶). For general background, see: Herrmann *et al.* (1995 ▶); Herrmann (2002 ▶); Littke & Fu (2002 ▶); Özdemir *et al.* (2005*c* ▶); Arduengo & Krafczyc (1998 ▶); Navarro *et al.* (2006 ▶). For related compounds, see: Liu *et al.* (2004 ▶); Therrien *et al.* (2004 ▶); Arslan *et al.* (2004*a* ▶,*b* ▶, 2005*a* ▶,*b* ▶, 2007*a* ▶,*b* ▶,*c* ▶). For puckering and asymmetry parameters, see: Cremer & Pople (1975 ▶); Nardelli (1983 ▶). For bond-length data, see: Allen *et al.* (1987 ▶).
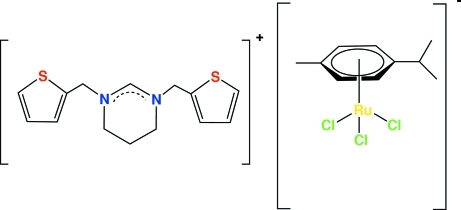


## Experimental

### 

#### Crystal data

(C_14_H_17_N_2_S_2_)[Ru(C_10_H_14_)Cl_3_]
*M* *_r_* = 619.05
Triclinic, 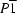

*a* = 9.989 (2) Å
*b* = 11.404 (2) Å
*c* = 12.922 (3) Å
α = 82.10 (3)°
β = 67.61 (3)°
γ = 72.59 (3)°
*V* = 1298.2 (6) Å^3^
*Z* = 2
Mo *K*α radiation
μ = 1.09 mm^−1^
*T* = 153 (2) K
0.24 × 0.12 × 0.07 mm

#### Data collection

Rigaku Mercury CCD diffractometer
Absorption correction: multi-scan (*REQAB*; Jacobson, 1998 ▶) *T*_min_ = 0.780, *T*_max_ = 0.928
9185 measured reflections
4606 independent reflections
4048 reflections with *I* > 2σ(*I*)
*R*_int_ = 0.020

#### Refinement

*R*[*F* ^2^ > 2σ(*F* ^2^)] = 0.032
*wR*(*F* ^2^) = 0.076
*S* = 1.12
4606 reflections
292 parameters
H-atom parameters constrained
Δρ_max_ = 0.61 e Å^−3^
Δρ_min_ = −0.58 e Å^−3^

### 

Data collection: *CrystalClear* (Rigaku/MSC, 2001 ▶); cell refinement: *CrystalClear*; data reduction: *CrystalClear*; program(s) used to solve structure: *SHELXTL* (Sheldrick, 2008 ▶); program(s) used to refine structure: *SHELXTL*; molecular graphics: *SHELXTL*; software used to prepare material for publication: *SHELXTL*.

## Supplementary Material

Crystal structure: contains datablocks global, I. DOI: 10.1107/S1600536808042773/hg2457sup1.cif

Structure factors: contains datablocks I. DOI: 10.1107/S1600536808042773/hg2457Isup2.hkl

Additional supplementary materials:  crystallographic information; 3D view; checkCIF report

## Figures and Tables

**Table 1:** Hydrogen-bond geometry (Å, °)

*D*—H⋯*A*	*D*—H	H⋯*A*	*D*⋯*A*	*D*—H⋯*A*
C1—H1⋯Cl1^i^	0.96	2.64	3.519 (4)	153
C1—H1⋯Cl2^i^	0.96	2.82	3.478 (4)	126
C10—H10*A*⋯Cl3^i^	0.96	2.75	3.654 (4)	156
C14—H14⋯Cl1^ii^	0.96	2.63	3.584 (4)	175

Symmetry codes: (i) 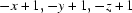; (ii) 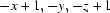.

**Fig. 1.**
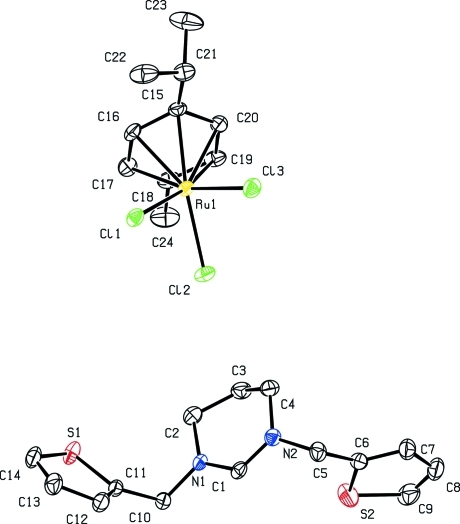
The molecular structure of the title compound, showing the atom-numbering scheme and displacement ellipsoids drawn at the 50% probability level.

**Fig. 2.**
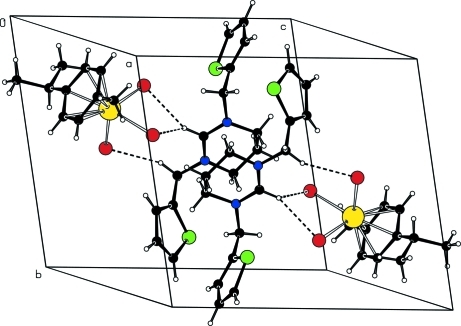
A packing diagram for (I).

**Fig. 3.**
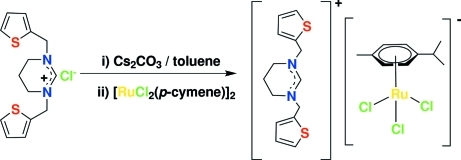
The formetion of the title compound.

## supplementary crystallographic information

### Comment

*N*-heterocyclic carbene ligands have emerged as one of the most important classes of compounds used for catalytic reactions such as Suzuki-Miyura, Sonogashira, Stille and Heck reactions (Herrmann *et al.*, 1995; Navarro *et al.*, 2006; Arduengo & Krafczyc, 1998; Herrmann, 2002; Littke & Fu, 2002). Recently, we have focused on the synthesis, characterization and application of palladium, platinum and ruthenium *N*-heterocyclic carbene complexes as catalysts (Yaşar *et al.*, 2008; Arslan *et al.*, 2007*a*, 2007*b*,2007*c*, 2004*a*, 2004*b*, 2005*a*, 2005*b*).

To continue our studies on this topic (Özdemir *et al.* (2005*a*, 2005*b*, 2005*c*, 2007, 2008)), we report herein the X-ray crystal structure of an *N*-heterocyclic carbene cation (1,3-*bis*(thiophen-2-ylmethyl)-3,4,5,6-tetrahydropyrimidinium) and trichloro(η^6^-*p*-cymene)ruthenat(II) anion compound. The molecular structure of the title compound, (I), is depicted in Fig. 1.

The structure of the title compound consists of [C_14_H_17_N_2_S_2_]^+^ cations and [C_10_H_14_Cl_3_Ru]^-^ anions. These groups are connected with four intermolecular C—H···Cl hydrogen bonds, thus forming a three-dimensional hydrogen-bonded network (Fig. 2). The intermolecular contacts are also listed in Table 1.

The thiophene rings are almost planar, while the pyrimidin ring is not planar. The deviations from planarity for the pyrimidin ring are C1 0.111 (4), N1 0.013 (3), C2 0.207 (4), C3 0.328 (4), C4 0.229 (4), and N2 0.010 (3) Å. The puckering parameters (Cremer & Pople, 1975) and the smallest displacement asymmetry parameters (Nardelli, 1983) for the pyrimidin ring are Q = 0.464 (4) Å, Θ = 55.1 (5) ° and φ = 236.6 (5)°, *q*_2_ = 0.381 (4) Å, and Δ*C*_2_(C1) = 2.8 (4), Δ*C*_s_(C1) = 66.6 (3). According to these results, the pyrimidin ring adopts an envelope conformation.

The coordination geometry of ruthenium is pseudooctahedral, with an average Ru—Cl bond distance of 2.427 Å. The ruthenium atom exhibits a distorted octahedral coordination with the benzene ring of the *p*-cymene ligand formally occupying three sites and three chloride atoms occupying three other sites. The distance between the centroid of the *p*-cymene ring and ruthenium atom is 1.972 (3) Å, which is longer than reported in other Ruthenium compounds (Liu *et al.*, 2004; Therrien *et al.*, 2004). All the other bond lengths in (I) are in normal ranges (Allen *et al.*, 1987).

Some C—N bond lengths (N1—C1 = 1.313 (4) Å and N2—C1 = 1.312 (4) Å) for the 1,3-*bis*(thiophen-2-ylmethyl)-3,4,5,6-tetrahydropyrimidinium cation are shorter than the average single C—N bond length of 1.48 Å, thus showing double bond character in these C—N bonds. The other C—N bond lengths (N1—C2 1.468 (4), N1—C10 1.475 (4), N2—C5 1.466 (4) and N2—C4 1.469 (4) Å) are in agreement with the expected 1.48 Å C—N single bond lengths. This information indicates a partial electron delocalization within the N1—C1—N2 fragment.

### Experimental

A suspension of 1,3-*bis*(thiophen-2ylmethyl)-3,4,5,6-tetrahydropyrimidinium chloride (1.1 mmol), Cs_2_CO_3_ (1.2 mmol) and [RuCl_2_(*p*-cymene)] (0.5 mmol) was heated under reflux in degassed toluene (20 ml) for 7 h. The reaction mixture was then filtered while hot, and the volume was reduced to about 10 ml before addition of *n*-hexane (15 ml) (Scheme 2). The precipitate formed was crystallized from CH_2_Cl_2_/diethylether (5:15 mL) to give complex as red-brown crystals. Yields: 0.232 g; 75%. *M*.p.: 235–236 *^o^*C. ^1^H NMR (CDCl_3_) δ: 1.38 (d, 6H, *J *= 6.9 Hz, CH_3_(C_6_H_4_)CH(C*H*_3_)_2_), 1.86 (quin., 2H, *J *= 6 Hz, NCH_2_C*H*_2_CH_2_N), 2.31 (s, 3H,*CH*_3_(C_6_H_4_)CH(CH_3_)_2_), 3.17 (m, 1H, CH_3_(C_6_H_4_)C*H*(CH_3_)_2_)_, _3.25 (t, 4H, *J *= 6 Hz, NC*H*_2_CH_2_C*H*_2_N), 4.75 (s, 4H, C*H*_2_C_4_H_3_S), 5.32 and 5.57 (d, 4H, *J *= 5.8 Hz, CH_3_(C_6_*H*_4_)CH(CH_3_)_2_), 7.05–7.64 (m, 6H, C_4_*H*_3_S), 8.91 (s, 1H, 2-CH). ^13^C NMR (CDCl_3_) δ: 19.0 (CH_3_(C_6_H_4_)CH(*C*H_3_)_2_), 19.1 (NCH_2_*C*H_2_CH_2_N), 22.3 (*C*H_3_(C_6_H_4_)CH(CH_3_)_2_), 30.8 (CH_3_(C_6_H_4_)*C*H(CH_3_)_2_), 42.3 (N*C*H_2_CH_2_*C*H_2_N), 54.5 (*C*H_2_C_4_H_3_S), 79.7, 81.8, 96.4 and 100.8 (CH_3_(*C*_6_H_4_)CH(CH_3_)_2_), 126.7, 127.5, 129.2 and 135.9 (*C*_4_H_3_S), 159.7 (2-*C*H). Anal. Calc. for C_24_H_31_S_2_N_2_RuCl_3_: C, 46.56; H, 5.05; N, 4.53%. Found: C, 47.19; H, 5.15; N, 4.71%.

### Crystal data

**Table:**

(C_14_H_17_N_2_S_2_)[Ru(C_10_H_14_)Cl_3_]	*Z* = 2
*M**_r_* = 619.05	*F*(000) = 632
Triclinic, *P*1	*D*_x_ = 1.584 Mg m^−^^3^
Hall symbol: -P 1	Mo *K*α radiation, λ = 0.71073 Å
*a* = 9.989 (2) Å	Cell parameters from 4238 reflections
*b* = 11.404 (2) Å	θ = 2.6–26.4°
*c* = 12.922 (3) Å	µ = 1.09 mm^−^^1^
α = 82.10 (3)°	*T* = 153 K
β = 67.61 (3)°	Rod, red
γ = 72.59 (3)°	0.24 × 0.12 × 0.07 mm
*V* = 1298.2 (6) Å^3^	

### Data collection

**Table:**

Rigaku Mercury CCD diffractometer	4606 independent reflections
Radiation source: Sealed Tube	4048 reflections with *I* > 2σ(*I*)
Graphite Monochromator	*R*_int_ = 0.020
Detector resolution: 14.6306 pixels mm^-1^	θ_max_ = 25.2°, θ_min_ = 2.6°
ω scans	*h* = −11→11
Absorption correction: multi-scan (*REQAB*; Jacobson, 1998)	*k* = −13→13
*T*_min_ = 0.780, *T*_max_ = 0.928	*l* = −12→15
9185 measured reflections	

### Refinement

**Table:**

Refinement on *F*^2^	Primary atom site location: structure-invariant direct methods
Least-squares matrix: full	Secondary atom site location: difference Fourier map
*R*[*F*^2^ > 2σ(*F*^2^)] = 0.032	Hydrogen site location: inferred from neighbouring sites
*wR*(*F*^2^) = 0.076	H-atom parameters constrained
*S* = 1.12	*w* = 1/[σ^2^(*F*_o_^2^) + (0.0324*P*)^2^ + 1.2338*P*] where *P* = (*F*_o_^2^ + 2*F*_c_^2^)/3
4606 reflections	(Δ/σ)_max_ < 0.001
292 parameters	Δρ_max_ = 0.61 e Å^−^^3^
0 restraints	Δρ_min_ = −0.58 e Å^−^^3^

### Special details

**Table:**

Geometry. All e.s.d.'s (except the e.s.d. in the dihedral angle between two l.s. planes) are estimated using the full covariance matrix. The cell e.s.d.'s are taken into account individually in the estimation of e.s.d.'s in distances, angles and torsion angles; correlations between e.s.d.'s in cell parameters are only used when they are defined by crystal symmetry. An approximate (isotropic) treatment of cell e.s.d.'s is used for estimating e.s.d.'s involving l.s. planes.
Refinement. Refinement of *F*^2^ against ALL reflections. The weighted *R*-factor *wR* and goodness of fit *S* are based on *F*^2^, conventional *R*-factors *R* are based on *F*, with *F* set to zero for negative *F*^2^. The threshold expression of *F*^2^ > σ(*F*^2^) is used only for calculating *R*-factors(gt) *etc*. and is not relevant to the choice of reflections for refinement. *R*-factors based on *F*^2^ are statistically about twice as large as those based on *F*, and *R*- factors based on ALL data will be even larger.

### Fractional atomic coordinates and isotropic or equivalent isotropic displacement parameters (Å^2^)

**Table:**

	*x*	*y*	*z*	*U*_iso_*/*U*_eq_	
Ru1	0.38371 (3)	0.30813 (2)	0.14309 (2)	0.01843 (9)	
Cl1	0.26136 (9)	0.23176 (6)	0.32961 (6)	0.02416 (17)	
Cl2	0.52125 (9)	0.38864 (7)	0.22412 (7)	0.02633 (18)	
Cl3	0.18305 (9)	0.49582 (7)	0.19463 (7)	0.02786 (18)	
C15	0.2964 (4)	0.2481 (3)	0.0344 (3)	0.0228 (7)	
C16	0.3788 (4)	0.1460 (3)	0.0795 (3)	0.0235 (7)	
H16	0.3314	0.0829	0.1191	0.028*	
C17	0.5297 (4)	0.1327 (3)	0.0687 (3)	0.0269 (7)	
H17	0.5828	0.0612	0.0998	0.032*	
C18	0.6015 (4)	0.2255 (3)	0.0118 (3)	0.0275 (7)	
C19	0.5197 (4)	0.3320 (3)	−0.0321 (3)	0.0263 (7)	
H19	0.5660	0.3965	−0.0692	0.032*	
C20	0.3713 (4)	0.3422 (3)	−0.0210 (3)	0.0241 (7)	
H20	0.3178	0.4142	−0.0514	0.029*	
C21	0.1370 (4)	0.2648 (3)	0.0438 (3)	0.0293 (7)	
H21	0.0836	0.3498	0.0579	0.035*	
C22	0.0538 (4)	0.1876 (4)	0.1380 (3)	0.0413 (9)	
H22A	0.0931	0.1026	0.1184	0.062*	
H22B	−0.0514	0.2140	0.1493	0.062*	
H22C	0.0673	0.1975	0.2057	0.062*	
C23	0.1396 (5)	0.2341 (5)	−0.0692 (3)	0.0540 (12)	
H23A	0.2007	0.2774	−0.1284	0.081*	
H23B	0.0391	0.2586	−0.0695	0.081*	
H23C	0.1808	0.1473	−0.0804	0.081*	
C24	0.7590 (4)	0.2154 (4)	0.0009 (3)	0.0415 (9)	
H24A	0.7889	0.1509	0.0503	0.062*	
H24B	0.7640	0.2918	0.0204	0.062*	
H24C	0.8253	0.1969	−0.0749	0.062*	
S1	0.88600 (10)	0.14274 (7)	0.52353 (8)	0.0311 (2)	
S2	0.82905 (10)	0.82421 (9)	0.36678 (9)	0.0379 (2)	
N1	0.7975 (3)	0.4626 (2)	0.4658 (2)	0.0205 (5)	
N2	0.6275 (3)	0.6428 (2)	0.4401 (2)	0.0217 (6)	
C1	0.7099 (3)	0.5733 (3)	0.4956 (3)	0.0212 (6)	
H1	0.7059	0.6053	0.5620	0.025*	
C2	0.8165 (4)	0.4094 (3)	0.3623 (3)	0.0306 (8)	
H2A	0.7505	0.3575	0.3790	0.037*	
H2B	0.9180	0.3600	0.3296	0.037*	
C3	0.7812 (4)	0.5106 (3)	0.2804 (3)	0.0287 (7)	
H3A	0.7792	0.4758	0.2177	0.034*	
H3B	0.8584	0.5531	0.2531	0.034*	
C4	0.6305 (4)	0.6005 (3)	0.3367 (3)	0.0259 (7)	
H4A	0.6144	0.6696	0.2870	0.031*	
H4B	0.5515	0.5614	0.3538	0.031*	
C5	0.5506 (4)	0.7712 (3)	0.4710 (3)	0.0251 (7)	
H5A	0.5365	0.7804	0.5475	0.030*	
H5B	0.4532	0.7926	0.4651	0.030*	
C6	0.6390 (4)	0.8577 (3)	0.3967 (3)	0.0236 (7)	
C7	0.5853 (4)	0.9710 (3)	0.3540 (3)	0.0299 (7)	
H7	0.4822	1.0059	0.3610	0.036*	
C8	0.7015 (5)	1.0309 (3)	0.2979 (3)	0.0361 (9)	
H8	0.6848	1.1114	0.2634	0.043*	
C9	0.8369 (5)	0.9630 (3)	0.2984 (3)	0.0396 (9)	
H9	0.9274	0.9892	0.2644	0.048*	
C10	0.8822 (4)	0.3889 (3)	0.5353 (3)	0.0223 (6)	
H10A	0.8744	0.4396	0.5919	0.027*	
H10B	0.9865	0.3610	0.4893	0.027*	
C11	0.8250 (3)	0.2799 (2)	0.5904 (3)	0.0202 (6)	
C12	0.7240 (4)	0.2726 (3)	0.6959 (3)	0.0283 (7)	
H12	0.6772	0.3394	0.7468	0.034*	
C13	0.6955 (4)	0.1548 (3)	0.7223 (3)	0.0329 (8)	
H13	0.6266	0.1340	0.7929	0.039*	
C14	0.7752 (4)	0.0756 (3)	0.6381 (3)	0.0315 (8)	
H14	0.7706	−0.0078	0.6417	0.038*	

### Atomic displacement parameters (Å^2^)

**Table:**

	*U*^11^	*U*^22^	*U*^33^	*U*^12^	*U*^13^	*U*^23^
Ru1	0.01972 (14)	0.01801 (13)	0.01928 (14)	−0.00846 (9)	−0.00606 (11)	−0.00118 (9)
Cl1	0.0309 (4)	0.0229 (4)	0.0195 (4)	−0.0138 (3)	−0.0051 (3)	−0.0002 (3)
Cl2	0.0284 (4)	0.0307 (4)	0.0263 (4)	−0.0151 (3)	−0.0100 (4)	−0.0045 (3)
Cl3	0.0270 (4)	0.0215 (4)	0.0339 (4)	−0.0038 (3)	−0.0104 (4)	−0.0048 (3)
C15	0.0270 (17)	0.0242 (15)	0.0205 (16)	−0.0072 (13)	−0.0104 (14)	−0.0056 (13)
C16	0.0302 (18)	0.0184 (14)	0.0228 (16)	−0.0097 (12)	−0.0065 (14)	−0.0052 (12)
C17	0.0283 (18)	0.0237 (15)	0.0237 (17)	−0.0007 (13)	−0.0061 (15)	−0.0085 (13)
C18	0.0225 (17)	0.0365 (18)	0.0220 (17)	−0.0069 (14)	−0.0030 (14)	−0.0128 (14)
C19	0.0278 (18)	0.0365 (17)	0.0169 (16)	−0.0164 (14)	−0.0036 (14)	−0.0040 (14)
C20	0.0305 (18)	0.0237 (15)	0.0200 (16)	−0.0098 (13)	−0.0097 (14)	0.0009 (13)
C21	0.0239 (18)	0.0356 (18)	0.0310 (19)	−0.0116 (14)	−0.0091 (15)	−0.0032 (15)
C22	0.034 (2)	0.066 (3)	0.032 (2)	−0.0290 (19)	−0.0075 (18)	−0.0043 (19)
C23	0.044 (3)	0.101 (4)	0.035 (2)	−0.040 (3)	−0.021 (2)	0.008 (2)
C24	0.0243 (19)	0.062 (2)	0.038 (2)	−0.0107 (17)	−0.0076 (18)	−0.0133 (19)
S1	0.0322 (5)	0.0171 (4)	0.0397 (5)	−0.0064 (3)	−0.0067 (4)	−0.0064 (3)
S2	0.0294 (5)	0.0416 (5)	0.0460 (6)	−0.0159 (4)	−0.0183 (4)	0.0163 (4)
N1	0.0258 (14)	0.0147 (11)	0.0224 (14)	−0.0056 (10)	−0.0095 (12)	−0.0022 (10)
N2	0.0220 (14)	0.0208 (12)	0.0227 (14)	−0.0070 (10)	−0.0077 (12)	0.0002 (11)
C1	0.0242 (16)	0.0193 (14)	0.0207 (16)	−0.0117 (12)	−0.0045 (14)	0.0001 (12)
C2	0.039 (2)	0.0231 (16)	0.0313 (19)	−0.0050 (14)	−0.0151 (17)	−0.0069 (14)
C3	0.0338 (19)	0.0314 (17)	0.0236 (17)	−0.0093 (14)	−0.0113 (16)	−0.0051 (14)
C4	0.0288 (18)	0.0279 (16)	0.0261 (17)	−0.0112 (13)	−0.0128 (15)	−0.0004 (14)
C5	0.0206 (16)	0.0211 (15)	0.0306 (18)	−0.0015 (12)	−0.0091 (15)	−0.0007 (13)
C6	0.0245 (17)	0.0241 (15)	0.0235 (17)	−0.0059 (13)	−0.0106 (14)	−0.0007 (13)
C7	0.036 (2)	0.0210 (15)	0.0309 (19)	−0.0052 (14)	−0.0125 (16)	0.0002 (14)
C8	0.058 (3)	0.0217 (16)	0.031 (2)	−0.0154 (16)	−0.0158 (19)	0.0032 (14)
C9	0.052 (3)	0.043 (2)	0.033 (2)	−0.0312 (19)	−0.0139 (19)	0.0075 (17)
C10	0.0242 (17)	0.0186 (14)	0.0259 (17)	−0.0070 (12)	−0.0108 (14)	0.0012 (12)
C11	0.0201 (16)	0.0152 (13)	0.0263 (17)	−0.0044 (11)	−0.0097 (14)	−0.0005 (12)
C12	0.036 (2)	0.0208 (15)	0.0283 (18)	−0.0104 (14)	−0.0104 (16)	−0.0008 (13)
C13	0.041 (2)	0.0276 (17)	0.0323 (19)	−0.0176 (15)	−0.0115 (17)	0.0066 (15)
C14	0.0322 (19)	0.0170 (15)	0.050 (2)	−0.0090 (13)	−0.0184 (18)	0.0012 (15)

### Geometric parameters (Å, °)

**Table:**

Ru1—C16	2.144 (3)	S2—C9	1.711 (4)
Ru1—C20	2.147 (3)	S2—C6	1.717 (3)
Ru1—C19	2.181 (3)	N1—C1	1.313 (4)
Ru1—C15	2.181 (3)	N1—C2	1.468 (4)
Ru1—C17	2.183 (3)	N1—C10	1.475 (4)
Ru1—C18	2.213 (3)	N2—C1	1.312 (4)
Ru1—Cl3	2.4185 (13)	N2—C5	1.466 (4)
Ru1—Cl1	2.4243 (11)	N2—C4	1.469 (4)
Ru1—Cl2	2.4377 (9)	C1—H1	0.9600
C15—C16	1.406 (4)	C2—C3	1.510 (5)
C15—C20	1.444 (4)	C2—H2A	0.9600
C15—C21	1.505 (4)	C2—H2B	0.9600
C16—C17	1.423 (5)	C3—C4	1.514 (5)
C16—H16	0.9600	C3—H3A	0.9600
C17—C18	1.418 (5)	C3—H3B	0.9600
C17—H17	0.9600	C4—H4A	0.9600
C18—C19	1.427 (5)	C4—H4B	0.9600
C18—C24	1.496 (5)	C5—C6	1.513 (4)
C19—C20	1.404 (5)	C5—H5A	0.9600
C19—H19	0.9600	C5—H5B	0.9600
C20—H20	0.9600	C6—C7	1.370 (4)
C21—C22	1.523 (5)	C7—C8	1.427 (5)
C21—C23	1.537 (5)	C7—H7	0.9600
C21—H21	0.9600	C8—C9	1.345 (6)
C22—H22A	0.9599	C8—H8	0.9600
C22—H22B	0.9599	C9—H9	0.9600
C22—H22C	0.9599	C10—C11	1.500 (4)
C23—H23A	0.9599	C10—H10A	0.9600
C23—H23B	0.9599	C10—H10B	0.9600
C23—H23C	0.9599	C11—C12	1.362 (5)
C24—H24A	0.9599	C12—C13	1.425 (4)
C24—H24B	0.9599	C12—H12	0.9600
C24—H24C	0.9599	C13—C14	1.347 (5)
S1—C14	1.718 (4)	C13—H13	0.9600
S1—C11	1.721 (3)	C14—H14	0.9600
			
C16—Ru1—C20	68.78 (12)	C21—C22—H22C	109.5
C16—Ru1—C19	81.44 (12)	H22A—C22—H22C	109.5
C20—Ru1—C19	37.86 (12)	H22B—C22—H22C	109.5
C16—Ru1—C15	37.94 (12)	C21—C23—H23A	109.5
C20—Ru1—C15	38.96 (11)	C21—C23—H23B	109.5
C19—Ru1—C15	69.75 (12)	H23A—C23—H23B	109.5
C16—Ru1—C17	38.37 (12)	C21—C23—H23C	109.5
C20—Ru1—C17	81.12 (13)	H23A—C23—H23C	109.5
C19—Ru1—C17	68.43 (13)	H23B—C23—H23C	109.5
C15—Ru1—C17	69.25 (12)	C18—C24—H24A	109.5
C16—Ru1—C18	68.65 (12)	C18—C24—H24B	109.5
C20—Ru1—C18	68.32 (13)	H24A—C24—H24B	109.5
C19—Ru1—C18	37.89 (13)	C18—C24—H24C	109.5
C15—Ru1—C18	81.99 (12)	H24A—C24—H24C	109.5
C17—Ru1—C18	37.63 (12)	H24B—C24—H24C	109.5
C16—Ru1—Cl3	127.04 (9)	C14—S1—C11	92.00 (16)
C20—Ru1—Cl3	86.08 (10)	C9—S2—C6	91.74 (18)
C19—Ru1—Cl3	106.63 (10)	C1—N1—C2	121.5 (3)
C15—Ru1—Cl3	94.62 (9)	C1—N1—C10	120.4 (3)
C17—Ru1—Cl3	163.87 (9)	C2—N1—C10	118.1 (2)
C18—Ru1—Cl3	143.21 (10)	C1—N2—C5	119.5 (3)
C16—Ru1—Cl1	87.76 (9)	C1—N2—C4	120.9 (3)
C20—Ru1—Cl1	142.92 (9)	C5—N2—C4	118.8 (3)
C19—Ru1—Cl1	166.75 (9)	N2—C1—N1	124.0 (3)
C15—Ru1—Cl1	105.94 (9)	N2—C1—H1	118.0
C17—Ru1—Cl1	98.33 (10)	N1—C1—H1	118.0
C18—Ru1—Cl1	130.29 (10)	N1—C2—C3	110.0 (3)
Cl3—Ru1—Cl1	86.00 (5)	N1—C2—H2A	109.7
C16—Ru1—Cl2	143.80 (9)	C3—C2—H2A	109.7
C20—Ru1—Cl2	128.81 (8)	N1—C2—H2B	109.7
C19—Ru1—Cl2	97.12 (9)	C3—C2—H2B	109.7
C15—Ru1—Cl2	166.84 (9)	H2A—C2—H2B	108.2
C17—Ru1—Cl2	107.40 (9)	C2—C3—C4	110.3 (3)
C18—Ru1—Cl2	88.00 (9)	C2—C3—H3A	109.6
Cl3—Ru1—Cl2	88.26 (4)	C4—C3—H3A	109.6
Cl1—Ru1—Cl2	87.05 (3)	C2—C3—H3B	109.6
C16—C15—C20	116.5 (3)	C4—C3—H3B	109.6
C16—C15—C21	123.8 (3)	H3A—C3—H3B	108.1
C20—C15—C21	119.6 (3)	N2—C4—C3	109.6 (3)
C16—C15—Ru1	69.62 (17)	N2—C4—H4A	109.8
C20—C15—Ru1	69.26 (17)	C3—C4—H4A	109.8
C21—C15—Ru1	130.4 (2)	N2—C4—H4B	109.8
C15—C16—C17	122.4 (3)	C3—C4—H4B	109.8
C15—C16—Ru1	72.44 (17)	H4A—C4—H4B	108.2
C17—C16—Ru1	72.29 (17)	N2—C5—C6	111.7 (3)
C15—C16—H16	118.8	N2—C5—H5A	109.3
C17—C16—H16	118.8	C6—C5—H5A	109.3
Ru1—C16—H16	129.0	N2—C5—H5B	109.3
C18—C17—C16	119.8 (3)	C6—C5—H5B	109.3
C18—C17—Ru1	72.33 (18)	H5A—C5—H5B	107.9
C16—C17—Ru1	69.34 (17)	C7—C6—C5	128.1 (3)
C18—C17—H17	120.1	C7—C6—S2	111.5 (2)
C16—C17—H17	120.1	C5—C6—S2	120.1 (2)
Ru1—C17—H17	130.9	C6—C7—C8	111.7 (3)
C17—C18—C19	119.2 (3)	C6—C7—H7	124.1
C17—C18—C24	121.1 (3)	C8—C7—H7	124.1
C19—C18—C24	119.7 (3)	C9—C8—C7	113.0 (3)
C17—C18—Ru1	70.04 (19)	C9—C8—H8	123.5
C19—C18—Ru1	69.82 (18)	C7—C8—H8	123.5
C24—C18—Ru1	130.8 (2)	C8—C9—S2	112.0 (3)
C20—C19—C18	119.8 (3)	C8—C9—H9	124.0
C20—C19—Ru1	69.78 (18)	S2—C9—H9	124.0
C18—C19—Ru1	72.29 (19)	N1—C10—C11	112.0 (2)
C20—C19—H19	120.1	N1—C10—H10A	109.2
C18—C19—H19	120.1	C11—C10—H10A	109.2
Ru1—C19—H19	130.4	N1—C10—H10B	109.2
C19—C20—C15	122.2 (3)	C11—C10—H10B	109.2
C19—C20—Ru1	72.36 (19)	H10A—C10—H10B	107.9
C15—C20—Ru1	71.78 (18)	C12—C11—C10	126.9 (3)
C19—C20—H20	118.9	C12—C11—S1	111.0 (2)
C15—C20—H20	118.9	C10—C11—S1	122.0 (2)
Ru1—C20—H20	129.6	C11—C12—C13	112.5 (3)
C15—C21—C22	113.6 (3)	C11—C12—H12	123.8
C15—C21—C23	108.2 (3)	C13—C12—H12	123.8
C22—C21—C23	110.5 (3)	C14—C13—C12	113.1 (3)
C15—C21—H21	108.1	C14—C13—H13	123.5
C22—C21—H21	108.1	C12—C13—H13	123.5
C23—C21—H21	108.1	C13—C14—S1	111.4 (2)
C21—C22—H22A	109.5	C13—C14—H14	124.3
C21—C22—H22B	109.5	S1—C14—H14	124.3
H22A—C22—H22B	109.5		
			
C20—Ru1—C15—C16	130.5 (3)	Cl3—Ru1—C18—C24	92.4 (4)
C19—Ru1—C15—C16	102.4 (2)	Cl1—Ru1—C18—C24	−76.4 (4)
C17—Ru1—C15—C16	28.64 (19)	Cl2—Ru1—C18—C24	8.0 (3)
C18—Ru1—C15—C16	65.3 (2)	C17—C18—C19—C20	−1.5 (5)
Cl3—Ru1—C15—C16	−151.54 (17)	C24—C18—C19—C20	−179.4 (3)
Cl1—Ru1—C15—C16	−64.39 (19)	Ru1—C18—C19—C20	−53.1 (3)
Cl2—Ru1—C15—C16	106.2 (4)	C17—C18—C19—Ru1	51.5 (3)
C16—Ru1—C15—C20	−130.5 (3)	C24—C18—C19—Ru1	−126.3 (3)
C19—Ru1—C15—C20	−28.11 (19)	C16—Ru1—C19—C20	66.25 (19)
C17—Ru1—C15—C20	−101.9 (2)	C15—Ru1—C19—C20	28.86 (18)
C18—Ru1—C15—C20	−65.2 (2)	C17—Ru1—C19—C20	103.8 (2)
Cl3—Ru1—C15—C20	77.93 (18)	C18—Ru1—C19—C20	132.3 (3)
Cl1—Ru1—C15—C20	165.08 (16)	Cl3—Ru1—C19—C20	−59.96 (18)
Cl2—Ru1—C15—C20	−24.3 (5)	Cl1—Ru1—C19—C20	102.0 (4)
C16—Ru1—C15—C21	117.6 (4)	Cl2—Ru1—C19—C20	−150.28 (17)
C20—Ru1—C15—C21	−111.8 (4)	C16—Ru1—C19—C18	−66.05 (19)
C19—Ru1—C15—C21	−139.9 (3)	C20—Ru1—C19—C18	−132.3 (3)
C17—Ru1—C15—C21	146.3 (3)	C15—Ru1—C19—C18	−103.44 (19)
C18—Ru1—C15—C21	−177.0 (3)	C17—Ru1—C19—C18	−28.52 (18)
Cl3—Ru1—C15—C21	−33.9 (3)	Cl3—Ru1—C19—C18	167.74 (15)
Cl1—Ru1—C15—C21	53.3 (3)	Cl1—Ru1—C19—C18	−30.3 (5)
Cl2—Ru1—C15—C21	−136.2 (3)	Cl2—Ru1—C19—C18	77.42 (17)
C20—C15—C16—C17	−2.0 (4)	C18—C19—C20—C15	0.3 (5)
C21—C15—C16—C17	179.7 (3)	Ru1—C19—C20—C15	−54.0 (3)
Ru1—C15—C16—C17	−54.6 (3)	C18—C19—C20—Ru1	54.3 (3)
C20—C15—C16—Ru1	52.6 (2)	C16—C15—C20—C19	1.4 (4)
C21—C15—C16—Ru1	−125.7 (3)	C21—C15—C20—C19	179.8 (3)
C20—Ru1—C16—C15	−30.84 (19)	Ru1—C15—C20—C19	54.2 (3)
C19—Ru1—C16—C15	−67.9 (2)	C16—C15—C20—Ru1	−52.8 (2)
C17—Ru1—C16—C15	−133.8 (3)	C21—C15—C20—Ru1	125.6 (3)
C18—Ru1—C16—C15	−105.0 (2)	C16—Ru1—C20—C19	−103.8 (2)
Cl3—Ru1—C16—C15	36.5 (2)	C15—Ru1—C20—C19	−133.9 (3)
Cl1—Ru1—C16—C15	119.80 (18)	C17—Ru1—C20—C19	−66.1 (2)
Cl2—Ru1—C16—C15	−158.27 (15)	C18—Ru1—C20—C19	−29.26 (18)
C20—Ru1—C16—C17	102.9 (2)	Cl3—Ru1—C20—C19	123.75 (18)
C19—Ru1—C16—C17	65.9 (2)	Cl1—Ru1—C20—C19	−158.17 (15)
C15—Ru1—C16—C17	133.8 (3)	Cl2—Ru1—C20—C19	39.2 (2)
C18—Ru1—C16—C17	28.82 (19)	C16—Ru1—C20—C15	30.09 (18)
Cl3—Ru1—C16—C17	170.29 (15)	C19—Ru1—C20—C15	133.9 (3)
Cl1—Ru1—C16—C17	−106.42 (19)	C17—Ru1—C20—C15	67.84 (19)
Cl2—Ru1—C16—C17	−24.5 (3)	C18—Ru1—C20—C15	104.7 (2)
C15—C16—C17—C18	0.8 (5)	Cl3—Ru1—C20—C15	−102.32 (18)
Ru1—C16—C17—C18	−53.8 (3)	Cl1—Ru1—C20—C15	−24.2 (3)
C15—C16—C17—Ru1	54.7 (3)	Cl2—Ru1—C20—C15	173.08 (14)
C16—Ru1—C17—C18	132.7 (3)	C16—C15—C21—C22	19.9 (5)
C20—Ru1—C17—C18	65.8 (2)	C20—C15—C21—C22	−158.3 (3)
C19—Ru1—C17—C18	28.70 (19)	Ru1—C15—C21—C22	−71.2 (4)
C15—Ru1—C17—C18	104.3 (2)	C16—C15—C21—C23	−103.1 (4)
Cl3—Ru1—C17—C18	103.7 (3)	C20—C15—C21—C23	78.7 (4)
Cl1—Ru1—C17—C18	−151.71 (18)	Ru1—C15—C21—C23	165.7 (3)
Cl2—Ru1—C17—C18	−62.2 (2)	C5—N2—C1—N1	−171.2 (3)
C20—Ru1—C17—C16	−66.9 (2)	C4—N2—C1—N1	−1.2 (4)
C19—Ru1—C17—C16	−104.0 (2)	C2—N1—C1—N2	3.4 (4)
C15—Ru1—C17—C16	−28.34 (18)	C10—N1—C1—N2	−177.8 (3)
C18—Ru1—C17—C16	−132.7 (3)	C1—N1—C2—C3	23.7 (4)
Cl3—Ru1—C17—C16	−29.0 (4)	C10—N1—C2—C3	−155.2 (3)
Cl1—Ru1—C17—C16	75.62 (18)	N1—C2—C3—C4	−50.8 (4)
Cl2—Ru1—C17—C16	165.13 (16)	C1—N2—C4—C3	−27.5 (4)
C16—C17—C18—C19	1.0 (5)	C5—N2—C4—C3	142.6 (3)
Ru1—C17—C18—C19	−51.4 (3)	C2—C3—C4—N2	52.6 (3)
C16—C17—C18—C24	178.8 (3)	C1—N2—C5—C6	97.4 (3)
Ru1—C17—C18—C24	126.4 (3)	C4—N2—C5—C6	−72.8 (3)
C16—C17—C18—Ru1	52.5 (3)	N2—C5—C6—C7	140.6 (3)
C16—Ru1—C18—C17	−29.34 (19)	N2—C5—C6—S2	−45.6 (4)
C20—Ru1—C18—C17	−104.1 (2)	C9—S2—C6—C7	0.7 (3)
C19—Ru1—C18—C17	−133.3 (3)	C9—S2—C6—C5	−174.1 (3)
C15—Ru1—C18—C17	−66.2 (2)	C5—C6—C7—C8	173.4 (3)
Cl3—Ru1—C18—C17	−153.21 (16)	S2—C6—C7—C8	−0.9 (4)
Cl1—Ru1—C18—C17	37.9 (2)	C6—C7—C8—C9	0.6 (5)
Cl2—Ru1—C18—C17	122.37 (19)	C7—C8—C9—S2	−0.1 (4)
C16—Ru1—C18—C19	104.0 (2)	C6—S2—C9—C8	−0.4 (3)
C20—Ru1—C18—C19	29.24 (17)	C1—N1—C10—C11	111.5 (3)
C15—Ru1—C18—C19	67.14 (18)	C2—N1—C10—C11	−69.6 (4)
C17—Ru1—C18—C19	133.3 (3)	N1—C10—C11—C12	−95.8 (4)
Cl3—Ru1—C18—C19	−19.9 (2)	N1—C10—C11—S1	85.4 (3)
Cl1—Ru1—C18—C19	171.28 (14)	C14—S1—C11—C12	0.0 (3)
Cl2—Ru1—C18—C19	−104.29 (17)	C14—S1—C11—C10	179.0 (3)
C16—Ru1—C18—C24	−143.7 (4)	C10—C11—C12—C13	−179.3 (3)
C20—Ru1—C18—C24	141.5 (4)	S1—C11—C12—C13	−0.3 (4)
C19—Ru1—C18—C24	112.3 (4)	C11—C12—C13—C14	0.6 (5)
C15—Ru1—C18—C24	179.4 (4)	C12—C13—C14—S1	−0.6 (4)
C17—Ru1—C18—C24	−114.4 (4)	C11—S1—C14—C13	0.3 (3)

### Hydrogen-bond geometry (Å, °)

**Table:**

*D*—H···*A*	*D*—H	H···*A*	*D*···*A*	*D*—H···*A*
C1—H1···Cl1^i^	0.96	2.64	3.519 (4)	153
C1—H1···Cl2^i^	0.96	2.82	3.478 (4)	126
C10—H10A···Cl3^i^	0.96	2.75	3.654 (4)	156
C14—H14···Cl1^ii^	0.96	2.63	3.584 (4)	175

Symmetry codes: (i) −*x*+1, −*y*+1, −*z*+1; (ii) −*x*+1, −*y*, −*z*+1.
